# Associations between temporal meal patterns, cardiovascular risk factors and cardiovascular disease in Swedish adults: cross-sectional and nested case-control analyses

**DOI:** 10.1007/s00394-026-04118-0

**Published:** 2026-09-24

**Authors:** Jenny Schultz, Lotta Moraeus, Sophie Hellstrand, Anna Karin Lindroos, Eva Warensjö Lemming

**Affiliations:** 1 Department of Food studies, Nutrition and Dietetics, Uppsala University, c/o BMC Intendenturen Box 570, SE-751 23 Uppsala, Sweden; 2 Division for Risk and Benefit Assessment, Swedish Food Agency, Uppsala, Sweden; 3 SciLifeLab Lund, Centre for Research Infrastructure in Health and Life Sciences, Lund University, Lund, Sweden; 4 Medical Epidemiology, Department of Surgical Sciences, Uppsala University, Uppsala, Sweden

**Keywords:** Chrononutrition, CVD, Breakfast skipping, Eating window, Meal frequency

## Abstract

**Purpose:**

Temporal meal patterns are suggested to influence cardiometabolic health, but existing evidence is limited, inconclusive, and largely cross-sectional. This study aimed to examine associations between temporal meal patterns and cardiovascular risk factors, and whether meal timing is associated with incident cardiovascular disease (CVD).

**Methods:**

The study included 3812 participants (18–75 years) in the Malmö Offspring Study who completed 4-day web-based dietary records and provided fasting blood samples. Cross-sectional analyses assessed associations between temporal meal patterns and risk factors, while a nested case–control study evaluated associations with incident CVD.

**Results:**

Meal frequency was nonlinearly associated with HDL-cholesterol, with the highest HDL levels observed at 4.7 meals/day. Each additional eating occasion was associated with 15% lower odds of metabolic syndrome (MS). Breakfast skipping was associated with higher total cholesterol (0.12 mmol/L 95% CI: 0.04, 0.21) and, among men, higher LDL-cholesterol (0.15 mmol/L 95% CI: 0.03, 0.26) but not with MS. Each additional hour of the eating window was associated with lower total and LDL-cholesterol (− 0.03 mmol/L 95% CI: − 0.05, − 0.01, and − 0.04, − 0.01) and a 7% (95% CI: 0.88, 0.98) reduction in odds of MS. A later first meal of the day, was associated with higher odds of MS (9% per hour, 95% CI: 1.01, 1.16). Prospectively, a later energy midpoint was associated with 31% higher odds of CVD (95% CI: 1.00, 1.74).

**Conclusion:**

Starting to eat early in the day, regular breakfast consumption, an earlier energy midpoint, higher meal frequency, and a longer eating window may be associated with favourable cardiometabolic health.

**Supplementary Information:**

The online version contains supplementary material available at https://doi.org/10.1007/s00394-026-04118-0.

## Introduction

Cardiovascular diseases (CVD) are the leading cause of death globally, with about 20.5 million deaths each year [1]. An unhealthy diet is one of the major risk factors for CVD, including underconsumption of healthy foods such as fruits, vegetables and whole grains, and overconsumption of unhealthy foods such as red meat, sugar-sweetened beverages and sodium [2]. In recent years, not only what we eat, but also when we eat has been suggested as a potential risk factor for cardiometabolic health [3].

Circadian rhythms are endogenous processes that operate in approximately 24 h cycles, driven by our biological clocks, primarily our master clock in the suprachiasmatic nucleus of the hypothalamus, as well as peripheral clocks [4, 5]. These clocks are influenced by external cues, called Zeitgebers, such as the light-dark cycle. The circadian rhythms regulate multiple biological functions including sleep-wake cycle, hormones, body temperature and metabolic functions. This means that our body is better prepared for food at certain times of the day, but also that food intake can serve as a cue and influence our circadian rhythms and metabolic processes. Research has suggested that eating in synchrony with the circadian rhythms may be favourable for our metabolic health. For example, eating at night has been associated with increased all-cause, cancer, and diabetes mortality [6].

Meal patterns such as skipping breakfast, late-night eating, and various fasting practices are all common time-related eating patterns. Previous research has shown an elevated risk for cardiovascular disease for those who skip breakfast compared to breakfast eaters [3, 7]. Eating late in the day is also suggested to be a risk factor for cardiovascular disease, and starting to eat early in the day appears to be beneficial for cardiometabolic health [8–10]. Also a greater meal frequency has been associated with improved cardiometabolic health, for example, lower concentrations of total- and low-density lipoprotein (LDL) cholesterol and lower risk for type 2 diabetes [3].

Although there appear to be associations between certain meal patterns and cardiovascular outcomes, no causal relationships have been established due to limited and inconclusive evidence, and most studies are cross-sectional [11]. There is also an inconsistency in definitions of meals, and in the methodology, such as whether to take energy intake, misreporting or other confounders into account. Therefore, further studies are needed to better understand these relationships, preferably with longitudinal data and using the proposed definitions of meals, which are all eating and drinking occasions with at least 210 kJ, and a minimum 15 min between two meals, and that the distinguishing between meal types should be up to the participant [3, 12].

The purpose of this study was to explore relationships between several temporal meal pattern characteristics, including meal frequency, timing of first and last meal of the day, eating window, energy midpoint and breakfast skipping, with cardiovascular risk factors in Swedish adults. In addition, we aimed to prospectively evaluate whether these temporal meal pattern characteristics were associated with incident cardiovascular disease. By examining multiple temporal eating behaviours using detailed time-stamped dietary records and relating these behaviours to both cardiovascular risk factors and disease outcomes within the same population, this study aims to provide a more comprehensive assessment of temporal eating patterns than has typically been available in previous studies.

## Methods

### Study design

We conducted two complementary analyses within the Malmö Offspring study. First, a cross-sectional analysis assessed associations between temporal meal patterns and cardiovascular risk factors at baseline. Second, a nested case–control study examined associations between temporal meal patterns and the incidence of cardiovascular disease during follow-up.

**Fig. 1:**
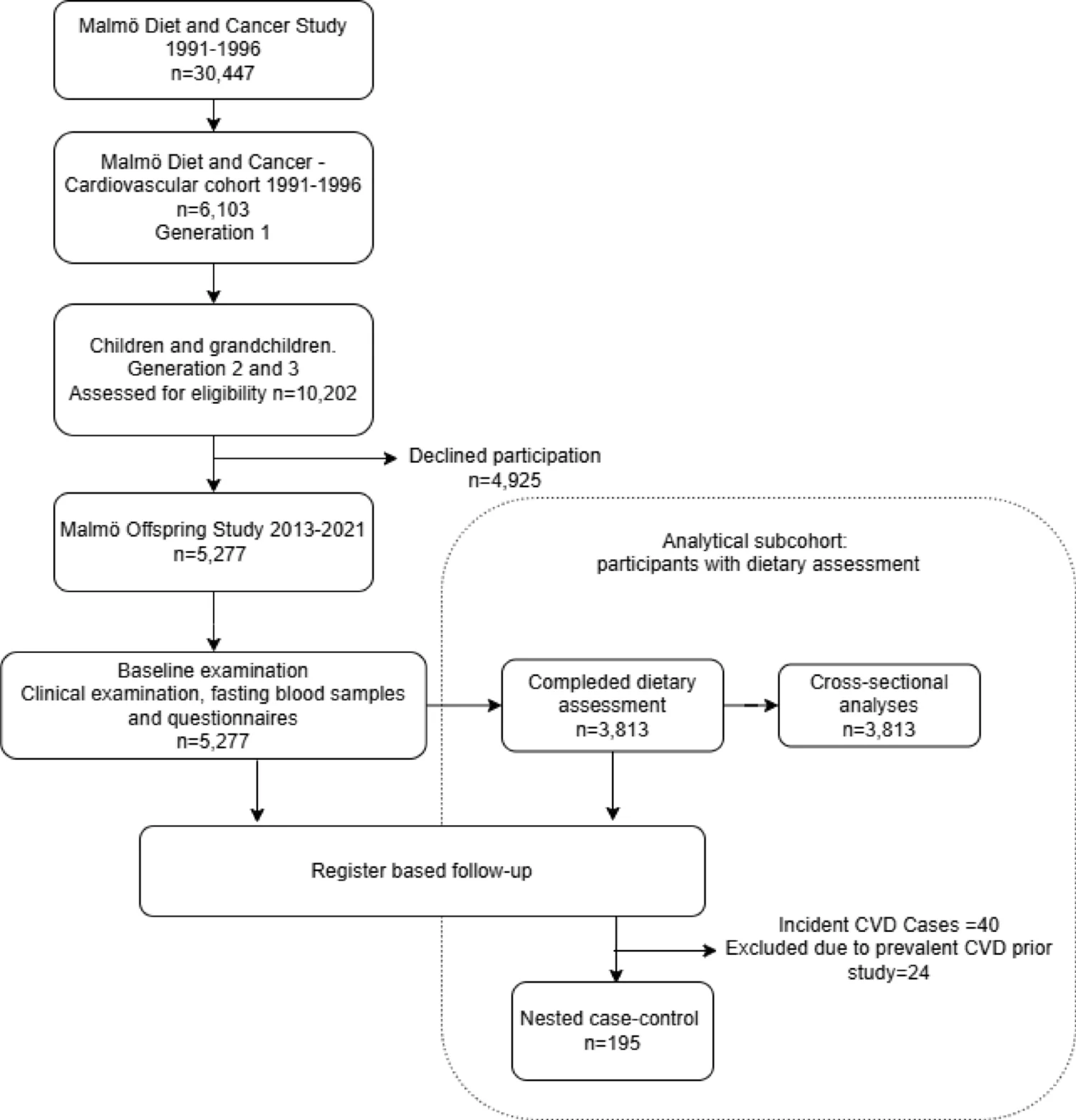
Flow chart of the selection of participants in the Malmö Offspring Study and the dietary analytical subcohort

### Study population

Malmö Offspring Study (MOS) started in 2013 and is a family-based cohort study of Swedish adults aged 18 to 75 years, with a register-based follow-up (Fig. 1) [13]. The participants of MOS consist of adult children and grandchildren (> 18 years) of participants in Malmö Diet and Cancer Study—the cardiovascular cohort study, conducted in the 1990s (*n* = 6103). Participants in MOS therefore form generation 2 and generation 3. A total of 10, 202 individuals were identified as potential participants for the cohort based on information from the Swedish Tax Agency and were invited to MOS. Exclusion criteria were age under 18 years, not living in southern Sweden (in Malmö until 2017) or difficulties in understanding information in Swedish. Participants visited the research clinic twice and underwent anthropometric measurements, blood sampling, completed questionnaires, cognitive testing, collected faeces, urine, and saliva samples, and completed a dietary assessment. Altogether, 5277 individuals participated in the MOS baseline examination, of which 3813 (72%) completed the on-line 4-day food record, an inclusion criterion for the present study. Follow-up data on morbidity and mortality in MOS were collected from the Swedish national registers until December 31, 2022.

Ethical approval for this study was obtained from the Regional Ethical Review Board in Lund (Dnr. 2012/594). All participants provided written informed consent prior to inclusion. The study was conducted in accordance with the ethical principles outlined in the Declaration of Helsinki regarding research involving human subjects.

### Dietary assessment

Participants were invited to complete web-based 4 day-food records, starting the day after the first visit to the clinic [14]. They also completed a short food frequency questionnaire, which was not used in the present study. The food records were completed using the validated, web-based, Riksmaten 2010 method, developed by the Swedish Food Agency (SFA) [15, 16] and used in the national dietary survey, Riksmaten adults 2010-11 [17]. Since participants started the registration the first day after the visit, all weekdays were represented in the study, and all participants had at least one weekend day included. The reported nutrient and energy intakes were calculated using the SFA food composition database version Riksmaten adults 2010-11. Participants selected pre-defined meal types Breakfast, Lunch, Dinner/evening meal and Other eating when entering meals in the food records, with at least 15 min between each meal. Images of portion sizes were embedded in the online tool for portion size estimation.

### Meal patterns

Meal patterns were derived from the food records, where a meal was defined as any eating occasion with at least 210 kJ [12]. No discrepancies were made between weekdays and weekends, and a mean value over the four days was used for all meal pattern variables for each participant. The same measures of meal patterns have previously been used in the Swedish population [18, 19].

Meal frequency was defined as the mean number of eating occasions per day. If the predefined meal type breakfast was not registered, the participants were identified as breakfast skippers. Those with no breakfast on any of the days were defined as complete skippers, and those with one to three missing breakfasts were irregular skippers. Both categories were grouped into breakfast skippers in most analyses, since complete skippers were too few for reaching enough power in the statistical analyses.

The clock time of the first and the last meal of the day was identified, and the eating window was defined as the number of hours between the first and the last meal of the day. The energy midpoint was defined as the time point of the day when 50% of the total daily energy intake had been consumed and indicates how a person distributes their energy intake throughout the day.

### Anthropometric and clinical measurements

Height and weight of the participants were measured at the clinic at baseline. Height was measured in cm with legs together looking straight ahead and weight was measured in kg using a calibrated balance beam or digital scale. Body mass index (BMI; kg/m^2^) was calculated based on these measurements. Resting blood pressure was measured and calculated as the mean of two readings in the supine position after a 10-minute rest, using an automatic device (Omron).

Fasting blood samples were collected on the first day of the visit, and metabolic biomarkers used in the present study were analysed by Department of Clinical Chemistry, Malmö. Total cholesterol (mmol/L), triglycerides (mmol/L), low-density lipoprotein (LDL) cholesterol (mmol/L), high-density lipoprotein (HDL) cholesterol (mmol/L), and fasting glucose (mmol/L) were analysed with standard methods and used as risk markers in the present analyses.

### Metabolic syndrome

Metabolic syndrome was defined according to the criteria of the International Diabetes Federation (IDF), with the exception that information on lipid-lowering medication was not available [20]. Thus, the definition in the present study included a waist circumference above 94 cm for men and 80 cm for women, in combination with at least two of the following: triglycerides ≥ 1.7 mmol/L, HDL cholesterol < 1.03 mmol/L for men and < 1.29 mmol/L for women, systolic blood pressure ≥ 130 mmHg or diastolic blood pressure ≥ 85 mmHg, or treatment for hypertension, and fasting glucose ≥ 5.6 mmol/L or diagnosed diabetes.

### Other variables

Information on physical activity, smoking, education level and medication use was collected from web-based questionnaires at baseline. Responses to the leisure physical activity question (sedentary leisure time; moderate physical activity during leisure time; moderate but regular physical activity during leisure time; regular physical activity and exercise) were categorised into a four-level ordinal variable reflecting physical activity. The question corresponds to the Saltin-Grimby Physical Activity Level Scale, a widely used and validated measure of leisure-time physical activity in Nordic population-based studies [21].

As education level participants described their highest level of education (no; primary; high school; university), which were used as a four-level ordinal variable. Medication use was in the present study categorised as use of prescribed medicine during the last week (yes; no). Smoking status was categorized according to the questionnaire responses (yes; never; former; sometimes).

Mean energy intake (MJ/day) was calculated for each participant, as well as the mean intake of fruit and vegetables (g/day) which aimed to serve as a proxy for a healthy diet. The amount of fruit and vegetable intake was completed by summarising recorded intakes in food categories of fruit and vegetables (including root vegetables but not potatoes) which included fresh, frozen, canned/conserved or fried food items.

To evaluate the accuracy of reported energy intake, misreporting was evaluated. Reported energy intake was compared with resting energy expenditure (REE), which was calculated based on equations by Henry [22]. The cut-offs for EI: REE were calculated with equations by Black, with physical activity level set to 1.55 [23]. We used this PAL since it is a commonly used default in the absence of measured total energy expenditure and because leisure-time physical activity in our sample was predominantly low to moderate. The lower limit was calculated to 1.02 and the upper limit to 2.35. Participants with EI: REE outside these cut-offs were identified as having implausible energy intakes (misreporters) but were not excluded from the study sample since it may lead to selection bias.

### Case-control sampling and matching procedure

Using endpoint data, incident cases of CVD occurring during the follow-up period were identified. CVD was defined as myocardial infarction (ICD I20-I25), stroke (ICD I60-I64), or heart failure (ICD I50) occurring between baseline and the final follow-up date (December 31, 2022). Individuals with prevalent CVD prior to study entry were excluded (*n* = 26).

A nested case-control study was conducted using risk-set sampling to account for varying entry times and follow-up durations. For each incident case of CVD, four controls were selected from the risk set of individuals who were still at risk (i.e., alive and free of CVD) at the time the case occurred. A ratio of 1:4 for cases and controls was chosen to optimise statistical power. Controls were matched to cases based on sex and 5-year age groups. In total, 40 incident CVD cases were identified (1%). Matching resulted in a final sample of 39 cases and 156 controls, in total 195 participants.

### Statistical analyses

Statistics were completed in STATA, version18 (StataCorp, College Station, Texas, USA). Variables were checked for normality, and variables with non-normal patterns were log-transformed before analysis (fasting glucose, triglycerides and HDL).

Associations between meal patterns and CVD risk markers were investigated with multivariable linear regression, with adjustment for age, sex, BMI, smoking, physical activity, education level, medication, energy intake and fruit and vegetable intake (proxy for healthy eating patterns). In models with fasting glucose as an outcome, participants with known diabetes were excluded. Associations between meal patterns and the risk of the metabolic syndrome were performed using multivariable logistic regression with adjustment for age, sex, BMI, smoking, physical activity, education level, medication, energy intake and fruit and vegetable intake (used as a proxy for healthy eating). The statistical analyses on CVD risk factors and metabolic syndrome were performed as complete case analyses, and depending on the outcome variable sample size ranged from 3498 to 3513 participants. Interaction terms between meal patterns and sex were tested, and where significant interactions were found, sex-stratified analyses were performed.

In the case-control analyses, missing values for the covariates smoking status (4%), physical activity (4%), education level (4%), and medication use (7%) were handled using multiple imputation by chained equations. The imputation model included age, sex, BMI, and other relevant covariates predictive of missingness. Twenty imputed datasets were generated and analysed separately, with results pooled using Rubin’s rules. The associations between meal patterns and risk of CVD were analysed using conditional logistic regression, adjusted for BMI, smoking, physical activity, education level, medication, energy intake, and fruit and vegetable intake.

Models were also tested for non-linear associations using squared terms and the likelihood ratio test. A sensitivity analysis was conducted to evaluate the impact of misreporting of energy intake on the estimates from the regression models by excluding misreporters from the analyses. To further assess the robustness of the findings, a sensitivity analysis excluding participants with any main meal skipping (breakfast, lunch or dinner) during the dietary recording period (*n* = 2009) was performed.

## Results

Out of 5260 participants included in MOS, 3813 (72%) completed the food records and were included in the present study. At baseline, age ranged from 18 to 75 years with a mean age of 42.8 years, and 54% were females. The mean follow-up time was 5.3 years (0.12–9.8 years). Baseline characteristics of the study populations are presented in Table 1.

**Table 1:** Baseline characteristics of study population, *n* = 3813

	Total study sample *n* = 3813	Females *n* = 2050	Males *n* = 1763	Case–control group *n* = 195	
				Cases *n* = 39	Controls *n* = 156
	Mean (SD)				
Age at baseline	42.8 (14.7)	42.6 (14.7)	43.0 (14.7)	55.1 (11.2)	54.9 (11.3)
BMI (kg/m^2^)	26.0 (4.7)	25.5 (5.0)	26.6 (4.2)	27.6 (5.2)	26.8 (4.2)
	n (%)				
Sex					
Females	2050 (54)	N/A	N/A	16 (41)	64 (41)
Males	1763 (46)	N/A	N/A	23 (59)	92 (59)
Smoking status					
No, never	2195 (58)	1155 (56)	1040 (59)	23 (66)	78 (51)
Former	973 (26)	560 (27)	413 (23)	6 (17)	59 (39)
Yes, sometimes	292 (8)	153 (8)	139 (8)	2 (6)	6 (4)
Yes, regularly	196 (5)	115 (6)	81 (5)	4 (11)	10 (7)
Missing	157 (4)	67 (3)	90 (5)	0	0
Leisure physical activity					
Sedentary	295 (8)	138 (7)	157 (9)	3 (9)	15 (10)
Moderate physical activity	1372 (36)	806 (39)	566 (32)	9 (26)	65 (43)
Moderate but regular physical activity	1081 (28)	583 (28)	498 (28)	11 (31)	47 (31)
Regular physical activity and exercise	898 (24)	451 (22)	447 (25)	12 (34)	26 (17)
Missing	167 (4)	72 (4)	95 (5)	0 (0)	0 (0)
Education level					
No	8 (0)	3 (0)	5 (0)	0 (0)	0 (0)
Primary	209 (6)	111 (5)	98 (6)	1 (3)	20 (13)
High school	2028 (53)	1006 (49)	1022 (58)	13 (37)	80 (52)
University	1403 (37)	856 (42)	547 (31)	21 (60)	53 (35)
Missing	165 (4)	74 (4)	91 (5)	0 (0)	0 (0)
Prescribed medicine, last week					
No	2206 (58)	1054 (51)	1152 (65)	17 (51)	77 (53)
Yes	1327 (35)	847 (41)	480 (27)	16 (48)	69 (47)
Missing	280 (7)	149 (7)	131 (7)	0 (0)	0(0)
Misreporting					
Under	1117 (29)	567 (28)	550 (31)	18 (46)	48 (31)
Over	17 (1)	9 (0)	8 (0)	0 (0)	1 (0)
Metabolic syndrome	678 (17)	272 (13)	406 (23)	15 (38)	34 (22)
	Mean (SD)				
Daily energy intake (MJ)	8.2 (2.6)	7.3 (2.0)	9.2 (2.7)	7.4 (1.7)	8.3 (2.6)
Total cholesterol (mmol/L)	4.9 (1.1)	5.0 (1.1)	4.9 (1.1)	5.6 (1.0)	5.2 (1.1)
HDL (mmol/L)	1.6 (0.5)	1.8 (0.5)	1.4 (0.4)	1.5 (0.5)	1.6 (0.4)
LDL (mmol/L)	3.1 (1.0)	3.1 (0.9)	3.3 (1.0)	3.8 (0.9)	3.4 (1.0)
Triglycerides (mmol/L)	1.1 (0.7)	1.0 (0.5)	1.3 (0.8)	1.4 (0.7)	1.2 (0.7)
Systolic blood pressure (mmHg)	116 (15)	112 (16)	120 (13)	129 (20)	122 (16)
Diastolic blood pressure(mmHg)	72 (10)	72 (10)	73 (10)	79 (10)	76 (9)
Fasting glucose (mmol/L)	5.4 (1.0)	5.3 (1.0)	5.4 (1.1)	5.6 (0.9)	5.4 (0.9)
Hba1c (mmol/mol)	34.6 (5.9)	34.3 (5.2)	35.0 (6.6)	38.7 (6.5)	35.6 (7.1)

BMI: body mass index, Leisure physical activity: To what extent have you engaged in physical activity and exercise during the past 12 months Misreporting: Based on cut-offs for EI: REE lower limit 1.02 upper limit 2.35. HDL: high-density lipoprotein cholesterol, LDL: low density lipoprotein cholesterol,

Metabolic syndrome: as defined by the International diabetes federation [20]

The average meal frequency was 4.2 meals per day with a slightly higher frequency for females (4.4) compared to males (4.1) (Table 2). Breakfast skipping was more common for males compared to females.

**Table 2:** Meal patterns of the total study population, separated by sex and for the case-control group

Meal pattern	Total study sample *n* = 3813	Females *n* = 2050	Males *n* = 1763	Case–control group *n* = 195	
				Cases *n* = 39	Controls *n* = 156
Mean (SD)					
Meal frequency	4.2 (1.0)	4.4 (1.0)	4.1 (1.1)	4.0 (1.1)	4.3 (1.1)
Eating window, (h)	11.3 (2.0)	11.4 (1.8)	11.3 (2.2)	11.3 (1.7)	11.5 (1.9)
Time of first EO	8:24 (1.6)	8:24 (1.5)	8:30 (1.8)	8:12 (1.5)	8:10 (1.3)
Time of last EO	19:48 (1.3)	19:46 (1.2)	19:48 (1.3)	19:31 (1.2)	19:38 (1.4)
Energy midpoint	15:30 (1.8)	15:29 (1.7)	15:36 (1.8)	15:49 (1.7)	15:19 (1.6)
n (%)					
Breakfast skipping	715 (19)	310 (15)	405 (23)	7 (18)	16 (10)
Complete skippers	64 (2)	24 (1)	40 (2)	1 (3)	1 (1)
Irregular skippers	651 (17)	286 (14)	365 (21)	6 (15)	15 (10)

Meal frequency: number of eating occasions per day. Eating window: hours between first and last eating occasion of the day. Breakfast skipping: Complete skipper did not register the meal type breakfast on any of the registration days, while irregular skippers did not registered breakfast on some of the registration days. Time of first EO: Clock time for the first eating occasion of the day. Time of last EO: Clock time for the last EO of the day. Energy midpoint: Clock time where 50% of the total energy intake is consumed

### Associations of meal patterns and CVD risk factors

Observed associations between temporal meal patterns and CVD risk factors are presented in Table 3. A non-linear, inverted U-shape association was observed between meal frequency and HDL cholesterol, with highest HDL around 4.7 meals per day, and lower values with lower and higher meal frequency (Supplemental Fig. 1).

**Table 3:** Associations between temporal meal patterns and cardiovascular risk factors, *n* = 3812

Meal pattern		Total cholesterol mmol/L		Triglycerides mmol/L		LDL mmol/L		HDL mmol/L		Systolic blood pressure mmHg		Diastolic blood pressure mmHg		Fasting glucose** mmol/L	
		β-coefficient (95% CI)	*P*-value	β-coefficient (95% CI)	*P*value	β-coefficient (95% CI)	*P*-value	β-coefficient (95% CI)	*P*-value	β-coefficient (95% CI)	*P*-value	β-coefficient (95% CI)	*P*-value	β-coefficient (95% CI)	*P*-value
Meal frequency	*Crude*	− 0.01 (− 0.05, 0.02)	0.44	− 0.05 (− 0.06, − 0.03)	<0.001	− 0.03 (− 0.06, − 0.00)	0.024	0.03 (0.02, 0.04)	<0.001	− 0.91 (− 1.39, − 0.43)	<0.001	− 0.44 (− 0.74, − 0.13)	0.005	− 0.00 (− 0.00, 0.01)	0.52
	*Adjusted*	− 0.03 (− 0.06, 0.01)	0.18	− 0.00 (− 0.02, 0.02)	0.98	− 0.02 (− 0.05, 0.02)	0.30	− 0.00* (− 0.01, 0.01)	0.81	-0.43 (− 0.92, 0.06)	0.088	− 0.24 (− 0.54, 0.06)	0.12	0.00 (− 0.01, 0.01)	0.76
Breakfast skipping ref breakfast eaters	*Crude*	− 0.05 (− 0.14, 0.04)	0.28	0.06 (0.02, 0.10)	0.004	− 0.00 (− 0.08, 0.08)	0.94	− 0.06 (− 0.08, − 0.04)	<0.001	− 0.44 (− 1.70, 0.83)	0.49	− 0.15 (− 0.95, 0.64)	0.71	− 0.00 (− 0.01, 0.01)	0.62
	*Adjusted*	0.12 (0.04, 0.21)	0.006	0.00 (− 0.03, 0.04)	0.83	0.08 (0.01, 0.16)	0.035	0.02 (− 0.00, 0.04)	0.11	0.50 (− 0.64, 1.65)	0.39	0.97 (0.28, 1.68)	0.007	− 0.00 (− 0.01. 0.01)	0.76
Eating window	*Crude*	− 0.01 (− 0.03, 0.01)	0.22	− 0.01 (0.02, − 0.01)	<0.001	− 0.01 (− 0.03, 0.00)	0.072	0.01 (0.01, 0.02)	<0.001	0.07 (− 0.17, 0.32)	0.55	− 0.02 (− 0.17, 0.14)	0.84	0.00 (0.00, 0.00)	0.036
	*Adjusted*	− 0.03 (− 0.0, − 0.01)	0.001	− 0.00 (− 0.01, 0.01)	0.31	− 0.03 (− 0.04, − 0.01)	0.001	0.00 (− 0.00, 0.01)	0.23	-0.10 (− 0.33, 0.13)	0.39	− 0.07 (− 0.21, 0.07)	0.34	0.00 (0.00, 0.01)	0.043
Time of first EO	*Crude*	− 0.04 (− 0.06, − 0.02)	0.001	0.01 (− 0.00, 0.02)	0.21	− 0.03 (− 0.04, − 0.01)	0.007	− 0.02 (− 0.02, − 0.01)	<0.001	− 0.67 (− 0.97, − 0.37)	<0.001	− 0.46 (− 0.65, − 0.27)	<0.001	− 0.00 (− 0.01, − 0.00)	0.006
	*Adjusted*	0.01 (− 0.01, 0.03)	0.19	0.01* (− 0.00, 0.01)	0.25	0.01 (− 0.02, 0.03)	0.28	− 0.00 (− 0.01, 0.00)	0.11	0.05 (− 0.22, 0.33)	0.71	0.04 (− 0.13, 0.21)	0.62	− 0.00 (− 0.00, 0.00)	0.29
Time of last EO	*Crude*	− 0.09 (− 0.11, − 0.06)	<0.001	− 0.03 (− 0.04, − 0.01)	<0.001	− 0.08 (− 0.10, − 0.06)	<0.001	0.0 (− 0.01, 0.01)	0.67	− 0.96 (− 1.35, − 0.57)	<0.001	− 0.83 (− 1.08, − 0.58)	<0.001	− 0.00 (− 0.00, 0.00)	0.77
	*Adjusted*	− 0.05* (− 0.08, − 0.02)	0.001	− 0.00 (− 0.01, 0.01)	0.94	− 0.05* (− 0.07, − 0.02)	<0.001	− 0.00* (− 0.01, 0.01)	0.83	− 0.16 (− 0.53, 0.21)	0.39	− 0.10* (− 0.32, 0.13)	0.39	0.00 (0.00, 0.01)	0.076
Energy midpoint	*Crude*	0.01 (− 0.01, 0.03)	0.50	0.01 (− 0.00, 0.02)	0.08	0.01 (− 0.01, 0.02)	0.56	− 0.00 (− 0.01, 0.00)	0.23	0.17 (− 0.10, 0.45)	0.22	0.14 (− 0.04, 0.31)	0.13	0.00 (0.00, 0.01)	0.043
	*Adjusted*	0.01 (− 0.01, 0.03)	0.15	0.00 (− 0.01, 0.01)	0.78	0.01 (− 0.01, 0.02)	0.46	0.00 (− 0.00, 0.01)	0.075	0.26 (0.01, 0.51)	0.040	0.21 (0.06, 0.36)	0.007	0.00* (− 0.00, 0.00)	0.30

Adjusted model: adjusted for age, sex, body mass index, physical activity (leisure time physical activity: sedentary leisure time, moderate physical activity during leisure time, moderate but regular physical activity during leisure time, regular physical activity and exercise, education level (no, primary, high school university), medication (prescribed medication during last week), smoking status (yes, never, former, sometimes), energy intake (mj/day), fruit and vegetable intake (grams/day). Meal frequency: For each additional eating occasion. Breakfast skipping: compared to breakfast eaters. Eating window: for each additional hour between the first and last meal of the day. Time of first EO: for each additional hour of the first eating occasion of the day. Time of last EO: for each additional hour of the last eating occasion of the day. Energy midpoint: for each additional hour of reaching 50% of daily energy intake

*Indicates that the exposure showed evidence of a non-linear association with the outcome in complementary analyses (e.g., when modelled with a quadratic term). Although linear regression estimates are presented here for consistency and comparability, non-linearity should be considered when interpreting these results

** Participant with prevalent diabetes were excluded from analyses

Skipping breakfast was associated with 0.12 mmol/L (95% CI: 0.04, 0.21) higher total cholesterol and 0.97 mmHg (95% CI: 0.28, 1.68) higher diastolic blood pressure compared to individuals who ate breakfast. Skipping breakfast was also associated with higher LDL in males (0.15 mmol/L; 95% CI: 0.03, 0.26) but not in females (*p* = 0.054 for the sex interaction). When evaluating complete breakfast skippers (*n* = 64) separately from irregular breakfast skippers, complete breakfast skippers showed stronger associations with cardiovascular risk factors than irregular breakfast skippers. Complete skippers had 0.28 mmol/L (95% CI: 0.02, 0.20) higher total cholesterol, 3.9 mmHg (95% CI: 0.42, 7.37) higher systolic blood pressure and 2.9 mmHg (95% CI: 0.78, 5.02) higher diastolic blood pressure compared to breakfast eaters. Although these stratified analyses should be interpreted with caution due to the low statistical power.

Each additional hour of eating window was associated with lower total cholesterol (− 0.03 mmol/L, 95% CI: − 0.05, − 0.01) and lower LDL cholesterol (− 0.03 mmol/L 95% CI: − 0.04, − 0.01). In addition, every one-hour delay in reaching the energy midpoint was associated with higher systolic (0.26 mmHg 95% CI: 0.01, 0.51) and diastolic blood pressure (0.21 mmHg 95% CI: 0.06, 0.36).

The time point of the last eating occasion showed a couple of inverse u-shaped non-linear relationships. For total cholesterol and LDL cholesterol, there were a positive association until around 18:00 when the highest values were observed, and thereafter a negative association (Supplemental Figs. 2 and 3). For HDL cholesterol, there was also a u-shaped association with the highest values when the last meal was consumed around 19:30 (Supplemental Fig. 4). For diastolic blood pressure, highest values were observed with the last meal around 19:00 (Supplemental Fig. 5).

**Fig. 2:**
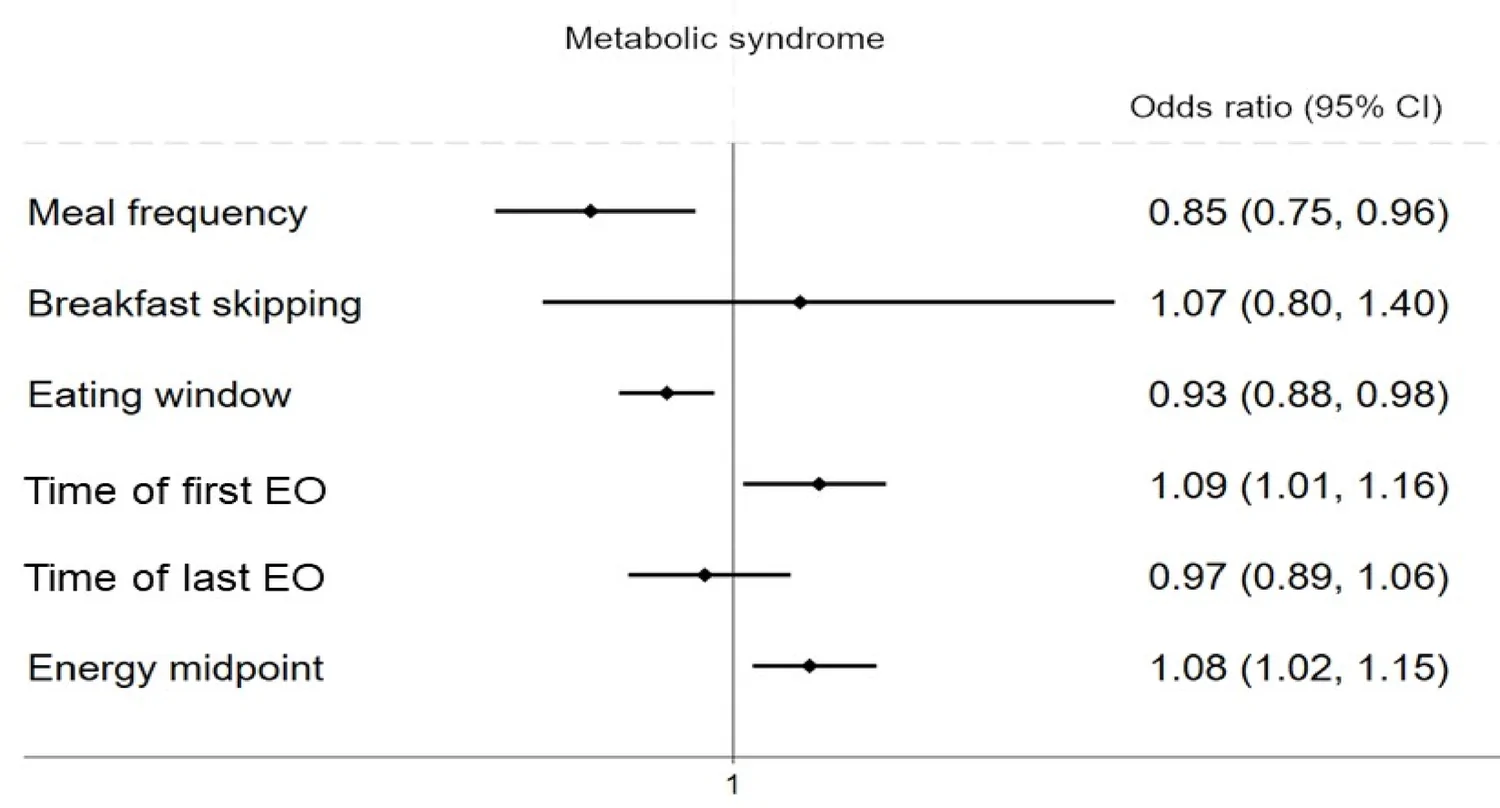
Associations between temporal meal patterns and the metabolic syndrome from adjusted models, expressed as Odds ratio and 95% confidence interval. Regression models adjusted for age, sex, BMI, physical activity, education level, smoking status, energy intake, fruit and vegetable intake. Metabolic syndrome was defined as by the International Diabetes Federation [20]; Waist circumference above 94 cm for men and 80 cm for women, in combination with at least two of the following: triglycerides ≥ 1.7 mmol/L, HDL cholesterol < 1.03 mmol/L for men and < 1.29 mmol/L for women, systolic blood pressure ≥ 130 mmHg or diastolic blood pressure ≥ 85 mmHg, or treatment for hypertension, and fasting glucose ≥ 5.6 mmol/L or diagnosed diabetes

Meal frequency: For each additional eating occasion. Breakfast skipping: compared to breakfast eaters. Eating window: for each additional hour between the first and last meal of the day. Time of first EO: for each additional hour of the first eating occasion of the day. Time of last EO: for each additional hour of the last eating occasion of the day. Energy midpoint: for each additional hour of reaching 50% of daily energy intake.

Meal patterns were also associated with the risk for the metabolic syndrome (Fig. 2). For each additional eating occasion, there was a lower odds ratio for the metabolic syndrome (OR 0.85 95% CI: 0.75–0.96). This relationship showed a weak non-linear relationship (likelihood ratio test *p* = 0.058) but was approximately linear across the observed range; therefore, the OR from the simpler model including only the linear term was used.

Also, for each additional hour in the eating window, the odds ratio for the metabolic syndrome decreased (OR 0.93 95% CI: 0.88–0.98) in the adjusted models. A higher odds ratio for metabolic syndrome was observed for each additional hour of the first meal of the day (OR 1.09, 95% CI: 1.01–1.16) as well as for every additional hour of the energy midpoint (OR 1.08, 95% CI: 1.02–1.15).

### Associations of meal patterns and risk of CVD

**Fig. 3:**
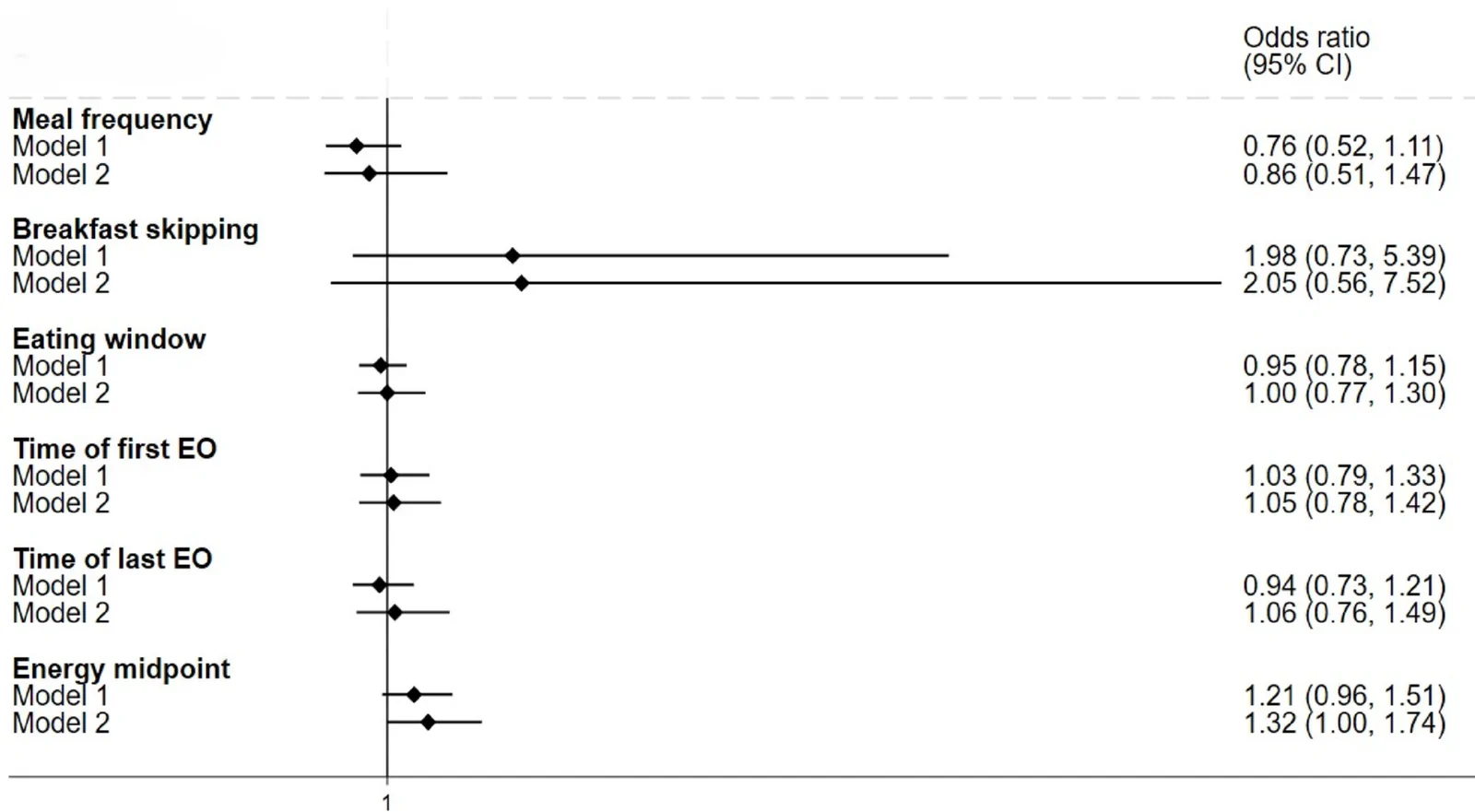
Pooled estimates for CVD risk in nested case-control (*n* = 195) by temporal meal patterns. Confidence intervals extending beyond the plotting range are truncated and indicated with arrows Model 1: Crude, Model 2: adjusted for physical activity, education level, medication, smoking status, energy intake, fruit and vegetable intake

In the nested case-control study, each additional later hour of energy midpoint was associated with a 32% higher risk for CVD (OR 1.32 95% CI 1.00–1.74), when adjusted for physical activity, education level, medication, smoking, energy intake and fruit and vegetable intake (Model 2) (Fig. 3). An elevated OR for CVD was observed for breakfast skipping in the adjusted models (OR 2.05, 95% CI: 0.56, 7.52), indicating a possible association. Adjustment for BMI in an additional regression model did not meaningfully change the effect estimates or their directions (data not shown). No other meal pattern was associated with the risk of CVD in this study sample.

Meal frequency: For each additional eating occasion. Breakfast skipping: compared to breakfast eaters. Eating window: for each additional hour between the first and last meal of the day. Time of first EO: for each additional hour of the first eating occasion of the day. Time of last EO: for each additional hour of the last eating occasion of the day. Energy midpoint: for each additional hour of reaching 50% of daily energy intake.

### Sensitivity analysis

To assess the robustness of the result against misreporting of energy intake, a sensitivity analysis where misreporters (*n* = 1134, 29.8%) were excluded was conducted. The findings showed that most effects retained their direction and magnitude, although some lost statistical significance, likely due to reduced statistical power. A few non-significant associations changed direction, which may indicate some sensitivity to the exclusion of misreporters, but these findings should be interpreted with caution, given the lack of statistical significance. In sensitivity analyses excluding participants with any main meal skipping (*n* = 2009), effect estimates remained similar in magnitude and direction to the primary analyses, although confidence intervals widened and associations were no longer statistically significant, likely reflecting reduced statistical power due to the substantially smaller sample size.

## Discussion

The current study examined how several temporal meal pattern variables associate with cardiovascular risk among Swedish adults. The results revealed that the following meal patterns—meal frequency, breakfast skipping, length of eating window, time point of first and last meal, and midpoint of daily energy intake—were all related to CVD risk and/or the metabolic syndrome. The prospective analyses in the case-control study revealed 31% higher odds for cardiovascular disease with a later energy midpoint and doubled odds with breakfast skipping (although not statistically significant). Because of the risk set sampling in the nested case-control study, the odds ratio can be interpreted as an approximation of hazard ratios.

Previous longitudinal research has associated breakfast consumption with a lower risk of metabolic syndrome [22], but this was not observed in our cross-sectional analysis. However, previous research has also shown associations between breakfast skipping and elevated total- and LDL cholesterol levels and higher blood pressure with similar effect sizes which are in line with the present results, but also associations with lower HDL levels [3, 24], which was not observed by us. Unfortunately, we had few complete breakfast skippers in the present study, but the stratified analyses with complete and irregular skippers showed stronger associations for complete skippers. Compliance with self-reported breakfast habits may also have influenced the observed associations, as participants who skipped reporting breakfast could have been misclassified as complete skippers, attenuating the true effect. Because of low statistical power, these stratified results should be interpreted with caution, but further studies should evaluate differences between complete and irregular breakfast skippers.

We observed a possible increased risk for cardiovascular disease associated with breakfast skipping, although the confidence interval was wide and included 1, indicating uncertainty. This tendency is consistent with associations reported in previous meta-analyses of cohort studies [7, 25], although our findings should be interpreted with caution due to limited sample size. It is still uncertain whether the benefits of eating breakfast stem from breaking the overnight fast, from the specific foods typically consumed at breakfast, or from its role as an indicator of a healthy lifestyle. Our analyses took lifestyle factors into account and used a proxy for a healthy diet, but other factors that were not included, such as working conditions, living situation or cohabiting, may also play a role. Previous authors have also cautioned against strong causal interpretations of observational associations between breakfast consumption and health outcomes, given the potential influence of residual confounding and lifestyle-related factors [26]. Furthermore, we could not observe any similar association between the related meal pattern variable, time of the first meal of the day, and CVD. This indicates that the beneficial effects of breakfast consumption involve more than simply the timing of the first meal.

In the current study, meal frequency was associated with HDL cholesterol levels in an inverted U-shaped relationship, but neither with total- nor LDL cholesterol levels. This is, in contrast with the results in the cross-sectional analysis undertaken in the EPIC (European Prospective Investigation into Cancer and Nutrition) Norfolk study. In that study with slightly older participants (45–75 years), associations were observed with total- and LDL cholesterol levels but not with HDL levels [27]. To note, that study did not evaluate non-linear relationships which seem to be important. A higher meal frequency was in the present study inversely associated with the metabolic syndrome (OR 0.85) and this is in line with the few studies previously published on this relationship [28–30]. Our study exhibited a slightly lower effect size than in the two cross-sectional studies from Asia [28, 30]. However, one of these studies did neither take energy intake nor healthy diet into account, and the other study reported significant results only for males adjusting for energy intake.

The findings regarding energy midpoint and the timing of the first meal are also consistent with previous research, which suggests that adopting an earlier eating pattern may be beneficial for both cardiometabolic outcomes and cardiovascular disease risk [8, 9]. The large longitudinal study by Palomar-Cros et al. found a hazard ratio of 1.06 for overall CVD for every 1 h later first meal [9]. The present study could only find associations with energy midpoint and possibly breakfast skipping, which are measures that also indicate an earlier eating timing. We did not, as mentioned, find any association between the timing of the first meal and CVD, but this variable was associated with the metabolic syndrome with a similar effect size (OR 1.09) in the cross-sectional analysis. Eating late or during nighttime has been associated with obesity and metabolic disorders as well as all-cause mortality and cancer [6, 31]. The present study did not observe an increased CVD risk with a later last meal. Instead, our findings support that later meals, after 18:00, are associated with lower total- and LDL cholesterol. This is supported by some other studies that found the same relationships [8]. The metabolic effects of these actual meal timings may be explained by the fact that the postprandial response of identical meals may differ depending on when they are consumed [32].

A longer eating window, in terms of hours between the first and the last meal of the day, appeared to have a potentially beneficial effect on total and LDL cholesterol levels, as well as being associated with 7% lower odds of metabolic syndrome with each additional hour. A 4 h longer eating window would be associated with a 0.12 mmol/L lower LDL and total cholesterol, these effect sizes are similar to a reduction of saturated fatty acids in the diet by 2 energy percent [33]. The length of eating windows has mainly been studied in the context of time-restricted eating (TRE), where shortened eating windows are promoted. Previous research has shown mixed results, with some studies suggesting benefits of a short eating window for obesity, type 2 diabetes, metabolic syndrome, and lipid and glucose metabolism [11, 34]. However, most randomised controlled trials in this field allow for energy reduction and weight loss, making it difficult to attribute effects solely to the length of the fasting period. Furthermore, much of this research has been conducted in populations with obesity or metabolic disturbances, such as type 2 diabetes or metabolic syndrome, limiting generalizability to healthy populations. In contrast, our study suggests that a longer eating window (i.e., a shorter fasting period) and also a higher meal frequency may be beneficial for cardiovascular risk factors and the metabolic syndrome in the general population, which challenges current evidence on TRE.

The prospective design, the detailed dietary assessment covering four days, and the number of participants are the main strengths of this study. The use of a nested case–control design improved analytic efficiency and contributed to stronger temporal validity and lower risk of bias. Although the cross-sectional design is a limitation, it is strengthened by combining it with longitudinal data, which allows for a more extensive evaluation of cardiovascular risk associated with meal timing. The present study also includes multiple measures of meal patterns, which allows for a more comprehensive understanding of their combined and independent effects. Some limitations need to be considered. The short follow-up time resulted in a limited number of CVD cases to include in the prospective analyses, leading to less robustness in the analyses. Even though we adjusted for many confounders, residual confounding cannot be ruled out. For example, information on sleep times, working conditions, further socioeconomic factors, or genetics was not considered, which may have affected these relationships. Dietary intake was self-reported and therefore subject to misreporting. Approximately 30% of participants underreported their energy intake relative to estimated resting energy expenditure. Although underreporting was evaluated in a sensitivity analysis, residual bias may remain. The cross-sectional part of the study has its limitation as we cannot draw causal conclusions. In addition, reverse causation cannot be ruled out, particularly in the cross-sectional analyses, as individuals with higher BMI or other cardiometabolic risk factors may have changed their meal patterns before study participation. Participants in MOS are recruited based on family ties to the previous cohort from the Malmö Diet and Cancer–Cardiovascular Cohort, which itself included a somewhat higher proportion of highly educated individuals and higher-level white-collar workers, as well as fewer current smokers and persons of non-Swedish origin. In addition, MOS is restricted to the southern region of Sweden (single-centre), which further limits the generalisability of the findings to the broader population. Not all participants in MOS completed the dietary assessment, and this may lead to further selection bias if these participants differ systematically from those who did not participate.

CVD is a multifactorial disease, and although we observed associations with meal patterns these factors likely explain only a small part of the overall risk. The effect sizes were modest, but this could still be important to further advance the understanding of the relationship between meal patterns and cardiovascular health and have implication on the population level. Prevention of cardiovascular disease is important and have major implications for public health. Research on meal patterns may contribute with easily conveyable messaging for the public. Future research should extend these findings, preferably with longitudinal data with longer follow-up times, in combination with a good quality of meal pattern assessment. Other meal pattern variables such as other meal skipping (i.e. lunch or dinner) or the combination of multiple exposures to the metabolic risk could also be further studied.

In conclusion, several meal patterns were associated with better cardiometabolic health including an early start to eating, eating breakfast, reaching the energy midpoint earlier in the day, higher eating frequency and a longer eating window.

## Supplementary Information

Below is the link to the electronic supplementary material.

Supplementary Material 1

## Data Availability

No datasets were generated or analysed during the current study.

## Abbreviations

MOS: Malmö Offspring study
SFA: Swedish food agency
IDF: International diabetes federation
REE: Resting energy expenditure
EI: Energy intake
