# Supplementary material for: Associations between temporal meal patterns, cardiovascular risk factors and cardiovascular disease in Swedish adults: cross-sectional and nested case-control analyses
Source: Eur J Nutr. 2026 Sep 24;65(7):264. doi: 10.1007/s00394-026-04118-0 (PMC13612618; doi:10.1007/s00394-026-04118-0)
Supplement: Supplementary file 1 [file 394_2026_4118_MOESM1_ESM.docx]

**Supplementary Figure 1.** Scatterplot of non-linear associations between HDL cholesterol and number of eating occasions.

**Supplementary Figure 2**. Scatterplot of non-linear associations between total cholesterol and time point of the last eating occasion.

**Supplementary Figure 3**. Scatterplot of non-linear associations between LDL cholesterol and time point of the last eating occasion.

**Supplementary Figure 4**. Scatterplot of non-linear associations between HDL cholesterol and time point of the last eating occasion.

**Supplementary Figure 5.** Scatterplot of non-linear associations between diastolic blood pressure and time point of the last eating occasion.
